# Impact of sleep apnea on Alzheimer's disease in relation to sex

**DOI:** 10.1002/alz70860_099705

**Published:** 2025-12-23

**Authors:** Sung Hoon Kang, Yu Jeong Park

**Affiliations:** ^1^ Korea University Guro Hospital, College of Medicine Korea University, Seoul, Korea, Republic of (South); ^2^ Korea University Guro Hospital, Seoul, Korea, Republic of (South)

## Abstract

**Background:**

We aimed to investigate the association between sleep apnea and incident dementia (dementia of the Alzheimer's type [DAT] and vascular dementia) and whether differences in the effects of sleep apnea on dementia depend on sex. Furthermore, we sought to determine whether obesity affects the sex‐specific relationship between sleep apnea and dementia.

**Method:**

We used de‐identified data on patients with sleep apnea and a control group aged ≥50 years from the Korean National Health Insurance Service. After propensity score matching to balance age and sex between the patient and control groups, 30,111 individuals with sleep apnea (patient group) and 121,528 individuals without sleep apnea (control group) were included. To investigate the impact of sleep apnea on the development of dementia, we used Cox proportional hazards regression after controlling for potential confounders.

**Result:**

Sleep apnea was predictive of developing DAT in both women (hazard ratio [HR]=1.30, 95% confidence interval [CI] 1.16‐1.44, *p* <0.001) and men (HR=1.13, 95% CI 1.03‐1.24, *p* = 0.012). The adverse effects of sleep apnea on DAT were more prominent in women than in men (*p* = 0.015 for sleep apnea×sex). Furthermore, obesity affected the sex‐specific relationship between sleep apnea and DAT. Specifically, the adverse effects of obese sleep apnea on the DAT were more pronounced in women than in men (*p* = 0.002 for obese sleep apnea×sex). In contrast, there were no differences in the effects of non‐obese sleep apnea on DAT between women and men (*p* = 0.667 for non‐obese sleep apnea×sex).

**Conclusion:**

Our results highlight sex differences in the adverse effects of sleep apnea on DAT. Furthermore, these results suggest that sex‐specific strategies for controlling sleep apnea are necessary to prevent DAT.

